# Supplementary material for: Modeling glioblastoma heterogeneity as a dynamic network of cell states
Source: Mol Syst Biol. 2021 Sep 16;17(9):e10105. doi: 10.15252/msb.202010105 (PMC8444284; doi:10.15252/msb.202010105)
Supplement: Supplementary file 5 — Source Data for Figure 3 [file MSB-17-e10105-s001.zip › Figure3A_sourcedata/GSEA_3065/nosigdb_state4.GseaPreranked.1622718430994/SUVA_NPC2_PMID_31327527.html]

Details for gene set SUVA\_NPC2\_PMID\_31327527[GSEA]

|  || Dataset | state4 |
| Phenotype | NoPhenotypeAvailable |
| Upregulated in class | na\_pos |
| GeneSet | SUVA\_NPC2\_PMID\_31327527 |
| Enrichment Score (ES) | 0.6556151 |
| Normalized Enrichment Score (NES) | 2.1483712 |
| Nominal p-value | 0.0 |
| FDR q-value | 1.7E-4 |
| FWER p-Value | 0.001 |
Table: GSEA Results Summary

  

Fig 1: Enrichment plot: SUVA\_NPC2\_PMID\_31327527      
 Profile of the Running ES Score & Positions of GeneSet Members on the Rank Ordered List

  

| PROBE | GENE SYMBOL | GENE\_TITLE | RANK IN GENE LIST | RANK METRIC SCORE | RUNNING ES | CORE ENRICHMENT || 1 | SOX4 |  |  | 18 | 0.402 | 0.1042 | Yes |
| 2 | TUBB2A |  |  | 61 | 0.287 | 0.1740 | Yes |
| 3 | NFIB |  |  | 75 | 0.269 | 0.2435 | Yes |
| 4 | MAP1B |  |  | 111 | 0.243 | 0.3028 | Yes |
| 5 | NFIX |  |  | 121 | 0.237 | 0.3647 | Yes |
| 6 | DDAH2 |  |  | 208 | 0.201 | 0.4046 | Yes |
| 7 | UCHL1 |  |  | 232 | 0.190 | 0.4515 | Yes |
| 8 | MLLT11 |  |  | 267 | 0.182 | 0.4945 | Yes |
| 9 | DPYSL3 |  |  | 298 | 0.174 | 0.5361 | Yes |
| 10 | TCF4 |  |  | 359 | 0.163 | 0.5700 | Yes |
| 11 | NDRG4 |  |  | 384 | 0.157 | 0.6081 | Yes |
| 12 | TAGLN3 |  |  | 531 | 0.133 | 0.6201 | Yes |
| 13 | CD24 |  |  | 636 | 0.121 | 0.6358 | Yes |
| 14 | NREP |  |  | 810 | 0.103 | 0.6355 | Yes |
| 15 | FNBP1L |  |  | 851 | 0.099 | 0.6556 | Yes |
| 16 | SEPT3 |  |  | 1113 | 0.081 | 0.6355 | No |
| 17 | DPYSL5 |  |  | 1949 | 0.048 | 0.5145 | No |
| 18 | CEP170 |  |  | 3307 | 0.018 | 0.3018 | No |
| 19 | RBFOX2 |  |  | 3386 | 0.016 | 0.2936 | No |
| 20 | KIF5C |  |  | 3632 | 0.008 | 0.2564 | No |
| 21 | BLCAP |  |  | 3639 | 0.008 | 0.2575 | No |
| 22 | DBN1 |  |  | 3829 | -0.000 | 0.2273 | No |
| 23 | MIAT |  |  | 4438 | -0.039 | 0.1402 | No |
| 24 | LBH |  |  | 5486 | -0.130 | 0.0071 | No |
| 25 | SOX11 |  |  | 5885 | -0.203 | -0.0025 | No |
| 26 | STMN1 |  |  | 5993 | -0.236 | 0.0433 | No |
Table: GSEA details [plain text format]

  

Fig 2: SUVA\_NPC2\_PMID\_31327527: Random ES distribution      
 Gene set null distribution of ES for **SUVA\_NPC2\_PMID\_31327527**
